# Can dexmedetomidine be a safe and efficacious sedative agent in post-cardiac surgery patients? a meta-analysis

**DOI:** 10.1186/cc11646

**Published:** 2012-09-27

**Authors:** Yi Yun Lin, Bin He, Jian Chen, Zhi Nong Wang

**Affiliations:** 1 Department of Cardiothoracic Surgery, Changzheng Hospital, the Second Military Medical University, Fengyang road 415#, Shanghai, 200003, China; 2 Department of Anesthesiology and SICU, Xinhua Hospital, Shanghai Jiaotong University School of Medicine, Kongjiang road 1665#, Shanghai, 200092, China

**Keywords:** Bradycardia, Hypotension, Delirium, Mechanical ventilation, Intensive care unit, Sedation

## Abstract

**Introduction:**

The aim of this study was to explore the use of dexmedetomidine as a safe and efficacious sedative agent in post-cardiac surgery patients.

**Methods:**

A systematic literature search of MEDLINE, EMBASE, the Cochrane Library and Science Citation Index until January 2012 and review of studies was conducted. Eligible studies were of randomized controlled trials or cohort studies, comparing dexmedetomidine with a placebo or an alternative sedative agent in elective cardiac surgery, using dexmedetomidine for postoperative sedation and available in full text. Two reviewers independently performed study selection, quality assessment, and data extraction.

**Results:**

The search identified 530 potentially relevant publications; 11 met selection criteria in this meta-analysis. Our results revealed that dexmedetomidine was associated with a shorter length of mechanical ventilation (mean difference -2.70 [-5.05, -0.35]), a lower risk of delirium (risk ratio 0.36 [0.21, 0.64]), ventricular tachycardia (risk ratio 0.27 [0.08, 0.97]) and hyperglycemia (risk ratio 0.78 [0.61, 0.99]), but may increase the risk of bradycardia (risk ratio 2.08 [1.16, 3.74]). But there was no significant difference in ICU stay, hospital stay, and morphine equivalents between the included studies. Dexmedetomidine may not increase the risk of hypotension, atrial fibrillation, postoperative nausea and vomiting, reintubation within 5 days, cardiovascular complications, postoperative infection or hospital mortality.

**Conclusions:**

Dexmedetomidine was associated with shorter length of mechanical ventilation and lower risk of delirium following cardiac surgery. Although the risk of bradycardia was significantly higher compared with traditional sedatives, it may not increase length of hospital stay and hospital mortality. Moreover, dexmedetomidine may decrease the risk of ventricular tachycardia and hyperglycemia. Thus, dexmedetomidine could be a safe and efficacious sedative agent in cardiac surgical patients.

## Introduction

Sedation, used to reduce stress response and provide anxiolysis [1], is an important component of postoperative management following cardiac surgery. The ideal sedative for use after cardiac surgery would have an immediate onset of action, be effective at providing immediate resolution of the agitation and anxiety, allow rapid recovery after discontinuation, lack drug accumulation, have minimal adverse effects, and be cost-effective [1,2]. However, no single agent or combination of agents has shown a clear superiority to meet these clinical standards [3].

Dexmedetomidine is a highly selective and potent central α_2_-receptor agonist which binds to transmembrane G protein-binding adrenoreceptors, and has no activity on the γ-aminobutyric acid (GABA) system [4]. By decreasing central nervous system sympathetic outflow, dexmedetomidine has analgesic effects known as opioid-sparing [5]. This property is unique among sedatives used in the intensive care unit (ICU) because it produces sedation and analgesia without causing respiratory depression [6]. In addition, the use of α_2_-agonists has been associated with lower cardiovascular complications in high-risk non-cardiac surgery [7].

Approved by the US Food and Drug Administration in 2008, dexmedetomidine represents only 4% of the drugs used for adult sedation outside of the operating room [8]. Currently, in Europe and the United States, benzodiazepines and propofol are the commonly used sedative agents in the ICU [3,9]. A recent systematic review stressed the use of dexmedetomidine as an alternative for postoperative sedation in critically ill adult patients [10]. The authors demonstrated that dexmedetomidine reduces the length of ICU stay compared with traditional sedative agents such as propofol, midazolam and morphine. From the clinician's viewpoint, dexmedetomidine has a favorable profile, as it can facilitate weaning from a mechanical ventilator by not depressing spontaneous ventilation. However, hypotension and bradycardia, the most common adverse effects of dexmedetomidine, have limited its use in the ICU. Concerns that these side effects could influence hemodynamic stability and increase hospital mortality have led to controversy regarding the benefits and risks of dexmedetomidine in postoperative sedation. Thus, the primary goal of the current study was to explore the use of dexmedetomidine as a safe and efficacious sedative agent following cardiac surgery.

## Materials and methods

### Search strategy and selection of studies

Two researchers independently carried out a comprehensive literature search. The literature search was conducted in January 2012 using multiple databases including EMBASE, MEDLINE, the Cochrane Library and Science Citation Index, from January 1979 through January 2012. A basic search was performed using keywords: 'dexmedetomidine' AND ('cardiac surgery' OR 'coronary artery bypass grafting' OR 'heart surgery' OR 'heart valve' OR 'cardiopulmonary bypass') AND ('sedation' OR 'sedative'). In addition, we reviewed abstracts from selected major cardiac surgical scientific meetings (American Heart Association, American Association for Thoracic Surgery, European Association for Cardio-Thoracic Surgery, and Asian Society for Cardiovascular Surgery) for unpublished studies, and contacted the authors for detailed information if needed. Our searches were restricted to English language studies for convenience reasons. To be eligible for inclusion in this article, publications met the following four inclusion criteria: (1) original research comparing dexmedetomidine with a placebo or an alternative sedative agent in elective cardiac surgery patients aged over 18 years; (2) study design: randomized controlled trial (RCT), non-randomized controlled trial or cohort study; (3) studies that continued use of dexmedetomidine for postoperative sedation for more than 6 hours, and not used for anesthesia in the operating theater, and (4) availability of full text (detailed information).

### Data abstraction and quality assessment

The two reviewers who extracted the data were blinded to the journal names and authors. If there were any differences in data abstraction or quality assessment, then the differences were reconciled by a third reviewer. The following information was abstracted and tabulated from each paper: author and year of publication; design; number of patients; surgery type; sedation goal and dose of dexmedetomidine, and placebo or alternative sedative agent. The following outcomes were extracted if reported: duration of mechanical ventilation; length of ICU stay; morphine equivalents; length of hospital stay; mortality at hospital discharge; risk of bradycardia or hypotension requiring interventions; risk of delirium; ventricular tachycardia; atrial fibrillation; hyperglycemia; nausea and vomiting; reintubation within 5 days after extubation, and any postoperative infection. If there was incomplete reporting of clinical outcomes in any of the articles, we attempted to contact the authors to obtain additional information. For example, given that virtually all patients were intubated for surgery, not all studies reported the mean value and standard deviation of the length of mechanical ventilation. We contacted authors [11-13] for additional detail on the outcomes mentioned above; however, no additional information was added. A ratio of relative risks and difference between treatment agents for categorical and continuous outcomes respectively, were extracted from all publications (if presented). Categorical outcomes are reported as risk ratio (RR) and corresponding 95% confidence interval (95% CI), while continuous outcomes are reported as weighted mean difference (MD). A *P*-value < 0.05 was used to determine statistical significance.

Quality assessment was undertaken independently by two authors (Table 1). The quality of included studies was evaluated based on a well established, validated Newcastle-Ottawa Scale (http://www.ohri.ca/programs/clinical_epidemiology/oxford.htm). Differences were resolved by discussion and consensus, and if disagreement still persisted, the opinions of all members of the research team were sought.

**Table 1:** Quality score of included studies

Study included		Newcastle-Ottawa Scale*								
		1	2	3	4	5A	5B	6	7	8
1	Herr (2003)	Yes	Yes	Yes	Yes	Yes	Yes	Yes	Yes	Yes
2	Anger (2010)	Yes	Yes	Yes	Yes	Yes	Yes	Yes	Yes	Yes
3	Yapici (2010)	Yes	Yes	Yes	Yes	No	Yes	Yes	Yes	Yes
4	Barletta (2009)	Yes	Yes	Yes	No	No	Yes	Yes	Yes	Yes
5	Maldonado (2009)	Yes	Yes	Yes	Yes	Yes	Yes	Yes	Yes	Yes
6	Dasta (2006)	Yes	Yes	Yes	No	No	No	No	Yes	Yes
7	Aziz (2011)	Yes	Yes	Yes	Yes	Yes	Yes	Yes	Yes	Yes
8	Shehabi (2009)	Yes	Yes	Yes	Yes	Yes	Yes	Yes	Yes	Yes
9	Wunsch (2010)	Yes	Yes	Yes	No	No	No	No	Yes	Yes
10	Corbett (2005)	Yes	Yes	Yes	Yes	Yes	Yes	Yes	Yes	Yes
11	Reichert (2011)^#^	Yes	Yes	Yes	Yes	Yes	Yes	Yes	Yes	Yes

*1 indicates exposed cohort truly representative; 2, non-exposed cohort drawn from the same community; 3, ascertainment of exposure; 4, outcome of interest not present as start of study; 5A, cohort comparable on basis of sedation goal level; 5B, cohort comparable on other factor(s); 6, quality of outcome assessment; 7, follow-up long enough for outcomes to occur; and 8, complete accounting for cohorts. ^#^For case-control study, 1 indicates cases independently validated; 2, cases are representative of population; 3, community control; 4, controls have no history of cardiac surgery; 5A, study control for sedation goal level; 5B, study controls for additional factor(s); 6, ascertainment of exposure by blinded interview or record; 7, same method of ascertainment used for cases and controls; and 8, non-response rate the same for cases and controls.

### Statistical analysis

Where outcomes of interest were reported by two or more studies, effect estimates were combined with meta-analyses in Review Manager (Version 5.1., The Cochrane Collaboration, 2011). Statistical heterogeneity between trials was evaluated by the Cochran χ^2 ^statistic, and was considered to be significant when the *P*-value for heterogeneity was ≤ 0.1. When statistical heterogeneity was observed, a random-effect model was used for the analysis. In the absence of statistically significant heterogeneity (*P*-value for heterogeneity > 0.1), only the fixed-effect model was utilized. Potential publication bias or small study bias was examined by visual inspection of constructed funnel plots.

To further test the robustness of the results, several sensitivity analyses were performed a priori. First, we evaluated whether the model of the statistical method (random-effect vs. fixed-effect model) would change the results; second, we determined whether the quality of publication, high quality (RCT) or low quality studies (retrospective cohort), could influence the results of the meta-analysis. Moreover, subgroup analysis was performed according to different criteria (for example, different sedative agents, high and low dose of dexmedetomidine). Where data were not presented in a way that could be included in the meta-analysis, or where only one study was identified for a given outcome, results of individual studies were presented.

## Results

### Included studies

**Figure 1:**
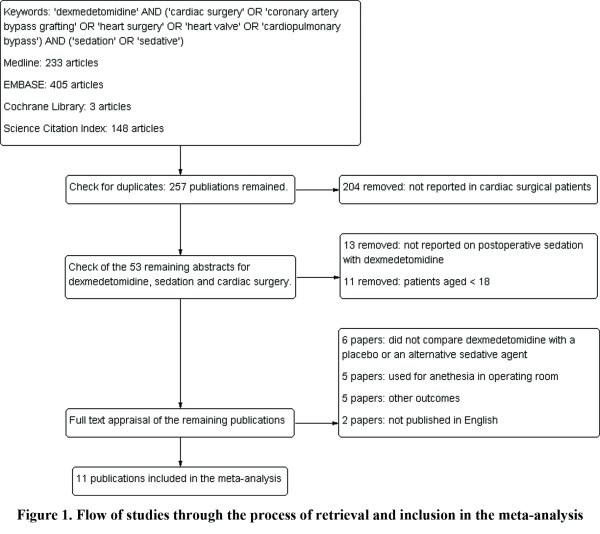
**Flow of studies through the process of retrieval and inclusion in the meta-analysis**.

A total of 530 studies were retrieved in the literature search, including 233 articles in MEDLINE, 146 articles in EMBASE, 3 articles in the Cochrane Library, and 148 articles in the Science Citation Index. After a check for duplicates and removal of reviews, 257 publications remained eligible for inclusion in the meta-analysis (Figure 1). Of these, the abstracts were screened for dexmedetomidine, sedation and cardiac surgery, and 204 publications were removed for non-cardiac surgery. Among the remaining 53 eligible studies, 13 publications were removed because the studies did not report on dexmedetomidine and postoperative sedation. Eleven papers were excluded because they included infant or pediatric cardiac surgery, six papers reported only the outcomes of dexmedetomidine and did not compare outcomes with a placebo or an alternative sedative agent, five papers were excluded because they reviewed the use of dexmedetomidine in the ICU but failed to report any outcomes, five were excluded for use of anesthesia in the operating theater, and two were excluded for not being published in English. Thus, 11 papers were included in the meta-analysis.

### Description of the included papers

Table 2 presents details of the included studies. Study designs included three randomized control trials, four prospective cohort studies and four retrospective studies. The smallest study contained 28 cardiac surgery patients (Aziz, *et al*.), whereas the largest study included 10,352 patients (Dasta, *et al*.). Eight studies compared dexmedetomidine with alternative hypnotic agents (seven studies with propofol [12-18], three with midazolam [16,17,19], and one with lorazepam [17]). Two studies compared dexmedetomidine with morphine [11,20]. The remaining study compared three drugs (dexmedetomidine, and propofol plus midazolam) with two drugs (propofol plus midazolam) [21].

**Table 2:** Characteristics of included studies

	First author (year of publication)	Study design	Patients, number	Surgery type	Sedation goal	**Dexmedetomidine**, infusion rate	Control, infusion rate
1	Herr (2003)	Randomized, open label study	295	CABG^§^	RSS^§ ^≥ 3 during assisted ventilation and ≥ 2 after extubation	1.0 ug/kg over 20 minutes then 0.2 to 0.7ug/kg/h to maintain	Propofol: not given
2	Anger (2010)	Prospective, descriptive study	56	Cardiac surgery	Similar RASS^§ ^between groups	0.6 ± 0.1 ug/kg/h	Propofol: 1.5 ± 0.6 ug/kg/h
3	Yapici (2010)	Prospective observational study	72	Cardiac surgery	Performing RASS scores at 48 and 60 h postoperative	0.3 to 0.7 ug/kg/h	Midazolam: 0.05 to 0.2 mg/kg/h
4	Barletta (2009)	Retrospective study	100	Cardiac surgery	Not given	0.3 ± 0.12 ug/kg/h	Propofol: 29 ± 11 ug/kg/min
5	Maldonado (2009)	RCT^§^	118	Cardiac surgery	RSS of 3 before extubation and 2 after extubation	loading dose: 0.4 ug/kg and then 0.2 to 0.7 ug/kg/h to maintain	Propofol: 25 to 50 ug/kg/min; midazolam: 0.5 to 2.0 mg/h
6	Dasta (2006)	Retrospective study	10352	Cardiac valve and vessel surgery	Not given	Three drugs (dexmedetomidine, propofol plus midazolam): not given	Two drugs (propofol plus midazolam): not given
7	Aziz (2011)	RCT	28	Cardiac surgery	Modified Ramsay Score and Numeric Pain Intensity Scale (compared within groups)	0.12 ± 0.03 ug/kg/h	Morphine: 13.2 ± 5.84 ug/kg/h
8	Shehabi (2009)	RCT	306	Pump cardiac surgery	Motor Activity Assessment Scale of 2 to 4	0.1 to 0.7 ug/kg/h	Morphine: 10 to 70 ug/kg/h
9	Wunsch (2010)	Retrospective cohort study	5332	CABG and valve surgery	Not given	Not given	Midazolam, lorazepam, propofol: not given
10	Corbett (2005)	Prospective randomized study	89	CABG	RSS of 5 for the first 2 h postoperative, followed by a score of 3 to 4 during intubation	Loading dose:1 ug/kg over 15 minutes, followed by 0.4 ug/kg/h	Propofol: 5 to 75 ug/kg/min
11	Reichert (2011)	Retrospective case-control study	70	CABG	SAS^§ ^targeted to scores of 3 or 4	0.3 to 0.7 ug/kg/h	Propofol: 15 to 30 ug/kg/min

CABG, coronary artery bypass grafting; RSS, Ramsay Sedation Score; RASS, Sedation-Agitation Scale; SAS, Sedation-Agitation Scale; RCT, randomized controlled trial.

### Outcomes of the pooled studies

**Figure 2:**
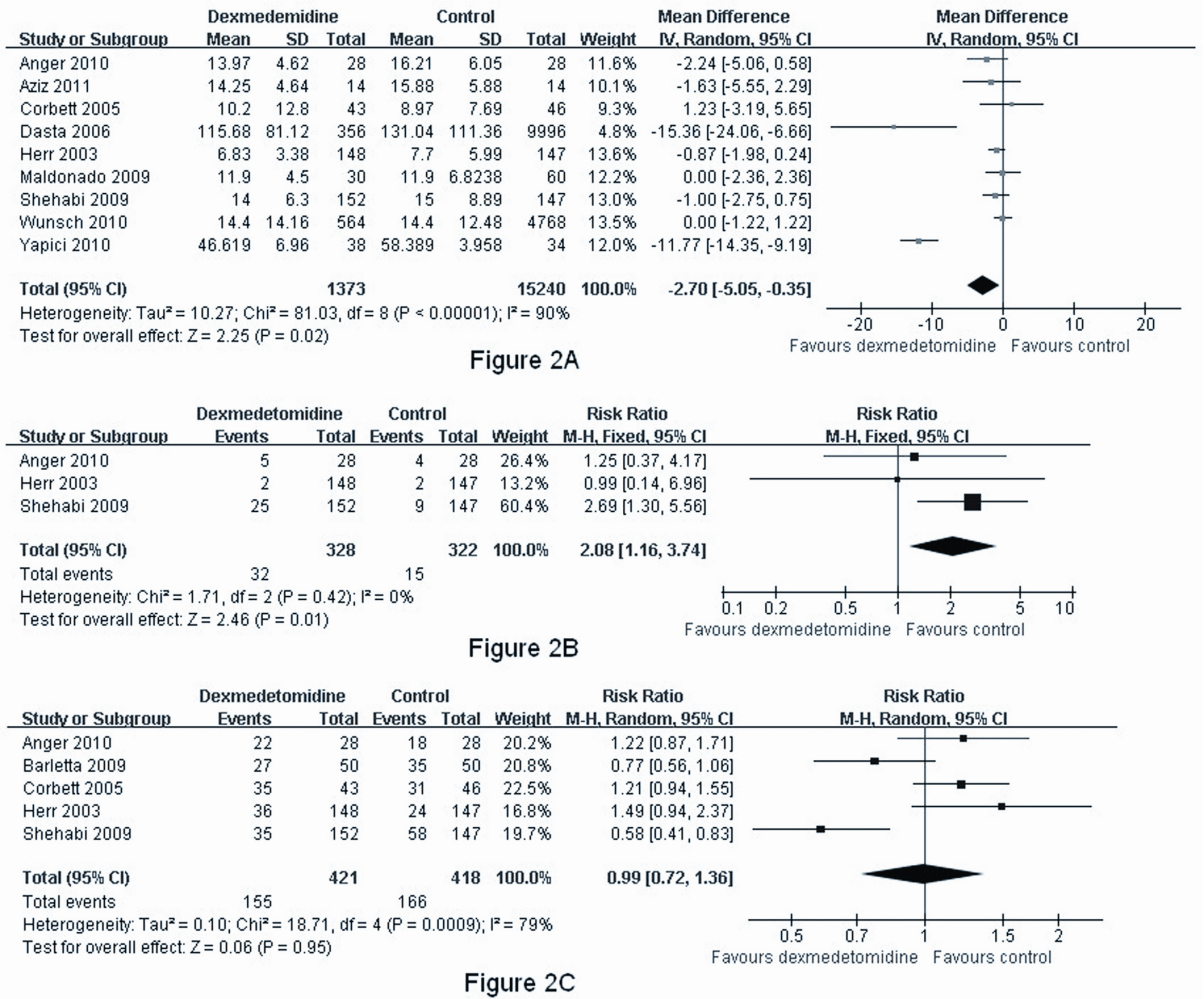
**Meta-analysis of postoperative mechanical ventilation, bradycardia and hypotension**. (A) Meta-analysis of length of mechanical ventilation (hours). (B) Meta-analysis of postoperative bradycardia. (C) Meta-analysis of postoperative hypotension.

Meta-analysis of nine studies [11,14-21] revealed that dexmedetomidine significantly reduced the length of mechanical ventilation (MD -2.70, 95% CI -5.05, -0.35, *P *= 0.02) (Figure 2A). Our results found dexmedetomidine treatment did not appear to reduce the length of ICU stay (MD -3.44, 95% CI -11.40, 4.52, *P *= 0.40) [15,16,18,20], length of hospital stay (MD -0.28, 95% CI -0.64, 0.07, *P *= 0.36) [15-17,20,21], or morphine equivalents (MD 0.45, 95% CI -1.86, 2.77, *P *= 0.70) [13,15,16,18] compared with other sedatives (Additional File 1). There was significant heterogeneity between the pooled studies in the length of mechanical ventilation, length of ICU stay and morphine equivalents. Thus, a random-effect model was used for these three analyses.

**Figure 3:**
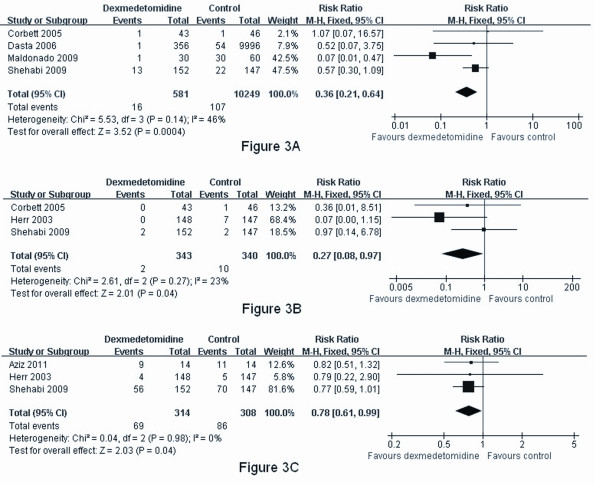
**Meta-analysis of postoperative delirium, ventricular tachycardia and hyperglycemia**. (A) Meta-analysis of postoperative delirium. (B) Meta-analysis of ventricular tachycardia. (C) Meta-analysis of hyperglycemia.

When pooled, dexmedetomidine was found to significantly increase the risk of bradycardia (RR 2.08, 95% CI 1.16, 3.74, *P *= 0.01) (Figure 2B), but not hypotension (RR 1.06, 95% CI 0.72, 1.56, *P *= 0.60) (Figure 2C). Additionally, dexmedetomidine reduced the incidence of delirium following cardiac surgery (RR 0.36, 95% CI 0.21, 0.64, *P *= 0.0004) (Figure 3A). Sedation with dexmedetomidine was associated with a lower risk of ventricular tachycardia (RR 0.27, 95% CI 0.08, 0.97, *P *= 0.04) (Figure 3B) and hyperglycemia (RR 0.78, 95% CI 0.61, 0.99, *P *= 0.04) (Figure 3C).

Dexmedetomidine was not associated with a significant reduction of atrial fibrillation (RR 0.90, 95% CI 0.62, 1.29, *P *= 0.56) [14,18,20], postoperative nausea and vomiting (RR 1.02, 95% CI 0.72, 1.46, *P *= 0.91) [11,14,20] (Additional File 2). Furthermore, there was no effect of dexmedetomidine on reintubation (RR 1.62, 95% CI 0.51, 5.13, *P *= 0.41) [12,15,20], postoperative infection (RR 0.92, 95% CI 0.65, 1.29, *P *= 0.62) [14,18,20] or hospital mortality (RR 0.89, 95% CI 0.38, 2.12, *P *= 0.08) [15,17,18,20,21] (Additional File 3). As to cardiovascular complications, Herr *et al*. [14] found no difference in the incidence of myocardial infarction (*P *= 0.371) and cardiac failure (*P *= 0.723) between dexmedetomidine and propofol, and Yapici *et al*. [19] and Shehabi *et al*. [20] reported similar incidence of postoperative low output syndrome (*P *= 0.093) and cardiac arrest (*P *= 0.513), respectively.

### Publication bias and sensitivity analyses

**Figure 4:**
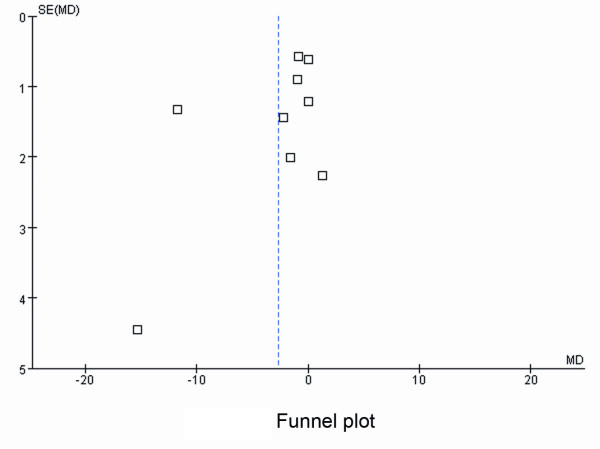
**Funnel plot with length of mechanical ventilation as an endpoint**. MD, mean difference; SE, Standard Error.

Using the length of mechanical ventilation as an endpoint, the funnel plot implies the possibility of publication bias (Figure 4). This bias could be explained by the following reasons: Barletta *et al*. [12] adopted a fast-track recovery model, which was different from other included articles, and it just needed shorter duration of mechanical ventilation; Yapici *et al*. [19] enrolled patients who had failed extubation and this caused longer-term ventilation support. The sensitivity analysis shows that regardless of which effect model was applied, the outcomes remained similar (Table 3). Further, we excluded the four retrospective studies [12,13,17,21] and Yapici *et al*.[19], which included the patients already presenting in a delirium state. Table 4 shows that after excluding the five above-mentioned studies, the outcomes still shared similarities with the outcomes when all eleven studies were included.

**Table 3:** Sensitivity analysis of outcomes according to the different effect models

Outcomes		Fixed-effect model	Random-effect model
Length of mechanical ventilation	MD	-1.39 (-2.04, -0.74)	-2.70 (-5.05, -0.35)
Duration in ICU	MD	-0.22 (-1.85, 1.41)	-3.44 (-11.40, 4.52)
Hospital stay	MD	-0.28 (-0.64, 0.07)	-0.36 (-0.83, 0.11)
Morphine equivalents	MD	1.11 (0.11, 2.11)	0.45 (-1.86, 2.77)
Hypotension	RR	0.94 (0.80, 1.10)	0.99 (0.72, 1.36)
Bradycardia	RR	2.08 (1.16, 3.74)	2.04 (1.12, 3.68)
Delirium	RR	0.36 (0.21, 0.64)	0.39 (0.13, 1.19)
Ventricular tachycardia	RR	0.27 (0.08, 0.97)	0.36 (0.07, 1.96)
Atrial fibrillation	RR	0.90 (0.62, 1.29)	0.88 (0.62, 1.27)
Hyperglycemia	RR	0.78 (0.61, 0.99)	0.78 (0.62, 0.99)
Vomiting and nausea	RR	1.02 (0.72, 1.46)	1.02 (0.71, 1.46)
Reintubation within 48 hours	RR	1.62 (0.51, 5.13)	1.51 (0.46, 4.96)
Any postoperative infection	RR	0.89 (0.38, 2.12)	0.89 (0.30, 2.64)
Hospital mortality	RR	0.79 (0.57, 1.09)	0.72 (0.37, 1.39)

MD: mean difference; RR: risk ratio. Values in parentheses are 95% CI.

**Table 4:** Subgroup analysis of outcomes according to study quality

Outcomes	Number of studies remaining	Results of remaining articles*	*P*-value for heterogeneity	*P*-value for overall effect
Length of mechanical ventilation	6	-0.87 (-1.67, -0.07)	0.78	0.03
Duration in ICU	4	-3.44 (-11.40, 4.52)	0.03	0.40
Hospital stay	3	-0.38 (-0.95, 0.19)	0.11	0.20
Morphine equivalents	3	1.25 (-0.98, 3.49)	0.06	0.27
Hypotension	4	1.06 (0.72, 1.56)	0.001	0.78
Bradycardia	3	2.08 (1.16, 3.74)	0.42	0.01
Delirium	3	0.35 (0.19, 0.63)	0.06	0.0005
Ventricular tachycardia	3	0.27 (0.08, 0.97)	0.27	0.04
Atrial fibrillation	3	0.90 (0.62, 1.29)	0.67	0.56
Hyperglycemia	3	0.78 (0.61, 0.99)	0.98	0.04
Vomiting and nausea	3	1.02 (0.72, 1.46)	0.48	0.91
Hospital mortality	3	1.00 (0.28, 3.60)	0.17	1.00
Any postoperative infection	3	0.89 (0.38, 2.12)	0.29	0.80
Reintubation within 48 hours	2	1.21 (0.33, 4.41)	/^#^	0.77

*Five studies excluded in this subgroup analysis: four [14,16,17,21] were retrospective studies and one [18] included the patients already presenting in a delirium state. ^/#^Heterogeneity was not applicable. Values in parentheses are 95% CI.

## Discussion

The current meta-analysis suggested that dexmedetomidine is associated with shorter length of mechanical ventilation, and lower risk of delirium, ventricular tachycardia and hyperglycemia following cardiac surgery, but that the risk of bradycardia was significantly higher compared with traditional sedative agents.

With greater clinical use and knowledge regarding α_2 _characteristic conscious sedation, a sedative agent without respiratory depression could hypothetically improve weaning and shorten extubation times. Our results showed that dexmedetomidine reduced the length of mechanical ventilation; however, there was no significant difference in the duration of ICU stay and hospital days following cardiac surgery. Similar to previous reports [10], we should interpret this result with caution, as there was significant heterogeneity between the pooled studies in the length of mechanical ventilation. This heterogeneity between the included studies could be attributed to different mechanical ventilation weaning protocols, different study designs or publication bias. Compared with commonly used sedatives, sedation with dexmedetomidine was not associated with higher risk of reintubation within 5 days after extubation. This meta-analysis provides evidence that with regard to duration of mechanical ventilation, cardiac surgery patients may benefit from the use of dexmedetomidine.

Our results also revealed that dexmedetomidine may decrease the incidence of delirium in cardiac surgery patients. The pathogenesis of delirium is not completely clear, but onset appears to be related to drug binding at the GABA receptor and release of deliriogenic mediators [2]. With high and specific receptor selectivity, dexmedetomidine does not bind to the GABA receptor and hence has intrinsic delirium-sparing effects, including asserting its sedative effects by blocking a single neurotransmitter, promoting cooperative sedation, producing sedation without respiratory depression and providing a more physiologic sleep-wake cycle. Moreover, it has been reported that dexmedetomidine administration causes the disappearance of delirium symptoms [19]. These reports are consistent with our study, suggesting that dexmedetomidine could be a favorable choice for the management of the delirium-state following cardiac surgery. Furthermore, because the dose of sedatives is determined by the level of sedation acquired, it would be essential to use mandatory daily interruption of sedation to avoid over-sedation. Daily interruption of sedation, especially early mobilization and fast-track weaning protocols, have been shown to decrease the incidence of delirium in cardiac surgery patients [22,23]. Thus, the use of dexmedetomidine, together with early mobilization and fast-track weaning protocols may provide additional benefit for patients following cardiac surgery.

The most frequently reported adverse events associated with dexmedetomidine treatment are bradycardia and hypotension [24]. In our study, use of dexmedetomidine was associated with increased risk of bradycardia, but was not accompanied by increased risk of systemic hypotension. Since the majority of the adverse events associated with dexmedetomidine administration occur during or shortly after the loading dose, it has been recommended that using a lower loading infusion rate during the first hour or eliminating the loading dose, may reduce the incidence of hypotension [14].

Previously, it has been shown that overdose of dexmedetomidine may cause first- or second-degree atrioventricular block [25]. Thus, caution should be used in sensitive patient populations, such as patients with left ventricular dysfunction or severe heart block, where the sympatholytic actions of α_2 _receptor agonists could prove especially dangerous. While severe bradycardia leading to cardiac arrest has been reported with the use of dexmedetomidine [26-28], the incidence of cardiovascular complications, including cardiac arrest after cardiac surgery, has not been shown to be significantly different compared with other sedatives. Further, our results showed that sedation with dexmedetomidine could reduce the risk of ventricular tachycardia compared with propofol and morphine. Cardiac conduction system dysfunction appears to be associated with use of dexmedetomidine [29]. Additional studies are warranted to investigate whether dexmedetomidine interferes with postoperative cardiac conduction and to underscore the value of adequate patient selection for the safe administration of dexmedetomidine.

It had been demonstrated that dexmedetomidine had opioid-sparing effects [30,31]. However, ICU sedation with dexmedetomidine did not reduce morphine equivalents in our analysis. Prior reports show that dexmedetomidine has no apparent effect on blood glucose concentration [4,32]. The current study reported the same outcome, that the use of dexmedetomidine decreases the risk of hyperglycemia after cardiac surgery. Since postoperative hyperglycemia is associated with increased in-hospital mortality in non-diabetic patients after cardiac surgery [33,34], this property may help the clinicians to better control plasma glucose levels after surgery.

However, there were some limitations in this meta-analysis. First, possible heterogeneity of study design, drugs, dosing regimens and the postoperative recovery unit model precluded meta-analysis of these study results. Also, the publication bias of some results, for example, length of mechanical ventilation, may affect the precision of this outcome. Second, difficulty maintaining consistency across studies is apparent when different goals for ideal sedation were adopted; for example, some studies required Ramsay level ≥ 3, while others used levels 2 to 4, 2 or 3, or 5. We highlight the need for a reliable and valid sedation scoring system to improve the interpretability of future studies. Thus, given that dexmedetomidine is currently much more expensive than commonly used drugs (for example, propofol), cost is becoming an increasingly critical factor in deciding whether to adopt new therapies. We were not able to compare cost in these trials because drug-related cost was not well-defined. Four, there was lack of long-term follow-up in patients treated with dexmedetomidine in the selected articles. Further studies are needed to explore the long-term effect of dexmedetomidine administration in cardiac surgery patients.

## Conclusions

In this meta-analysis, the most striking finding was that sedation with dexmedetomidine is associated with shorter length of mechanical ventilation and lower risk of delirium following cardiac surgery. Although the risk of bradycardia was significantly higher compared with traditional sedative agents, bradycardia may not increase the length of hospital stay and mortality at hospital discharge. Moreover, dexmedetomidine may decrease the risk of postoperative ventricular tachycardia and hyperglycemia. Thus, dexmedetomidine could be a safe and efficacious sedative agent in cardiac surgery patients. Further studies should underline the value of adequate patient selection for the safe use of dexmedetomidine following cardiac surgery.

## Key Messages

1. Dexmedetomidine is a highly selective and potent central α_2_-receptor agonist that produces sedation without causing respiratory depression, which is unique among sedatives used in the ICU. However, controversies exit in regarding the benefits and risks of dexmedetomidine in postoperative sedation.

2. Use of dexmedetomidine was found to be associated with shorter length of mechanical ventilation and lower risk of delirium following cardiac surgery.

3. Although the risk of bradycardia was significantly higher compared with traditional sedatives, dexmedetomidine may not increase the length of hospital stay and hospital mortality. Moreover, it may decrease the risk of ventricular tachycardia and hyperglycemia.

4. Dexmedetomidine could be a safe and efficacious sedative agent in cardiac surgery patients.

## Abbreviations

GABA: γ-aminobutyric acid; ICU: intensive care unit; RCT: randomized controlled trial; RR: risk ratio; CI: confidence interval; MD: mean difference.

## Competing interests

The authors declare that they have no competing interests.

## Authors' contributions

YL designed and performed searches, extracted data and wrote the manuscript draft. BH researched and wrote the discussion section and worked on manuscript revisions. JC performed searches and assisted with data extraction for the main meta-analysis. ZW conceived the study and helped with manuscript revisions. All authors read and approved the final manuscript.

## Supplementary Material

Additional File 1 **Figure showing meta-analysis of (A) ICU stay, (B) hospital stay, and (C) morphine equivalents**. Click here for file

Additional File 2 **Figure showing meta-analysis of (A) atrial fibrilation, and (B) vomiting and nausea**. Click here for file

Additional File 3 **Figure showing meta-analysis of (A) reintubation, (B) postoperative infection, and (C) hospital mortality**. Click here for file
