# Supplementary figures and images for: Can dexmedetomidine be a safe and efficacious sedative agent in post-cardiac surgery patients? a meta-analysis
Source: Crit Care. 2012 Sep 27;16(5):R169. doi: 10.1186/cc11646 (PMC3682268; doi:10.1186/cc11646)

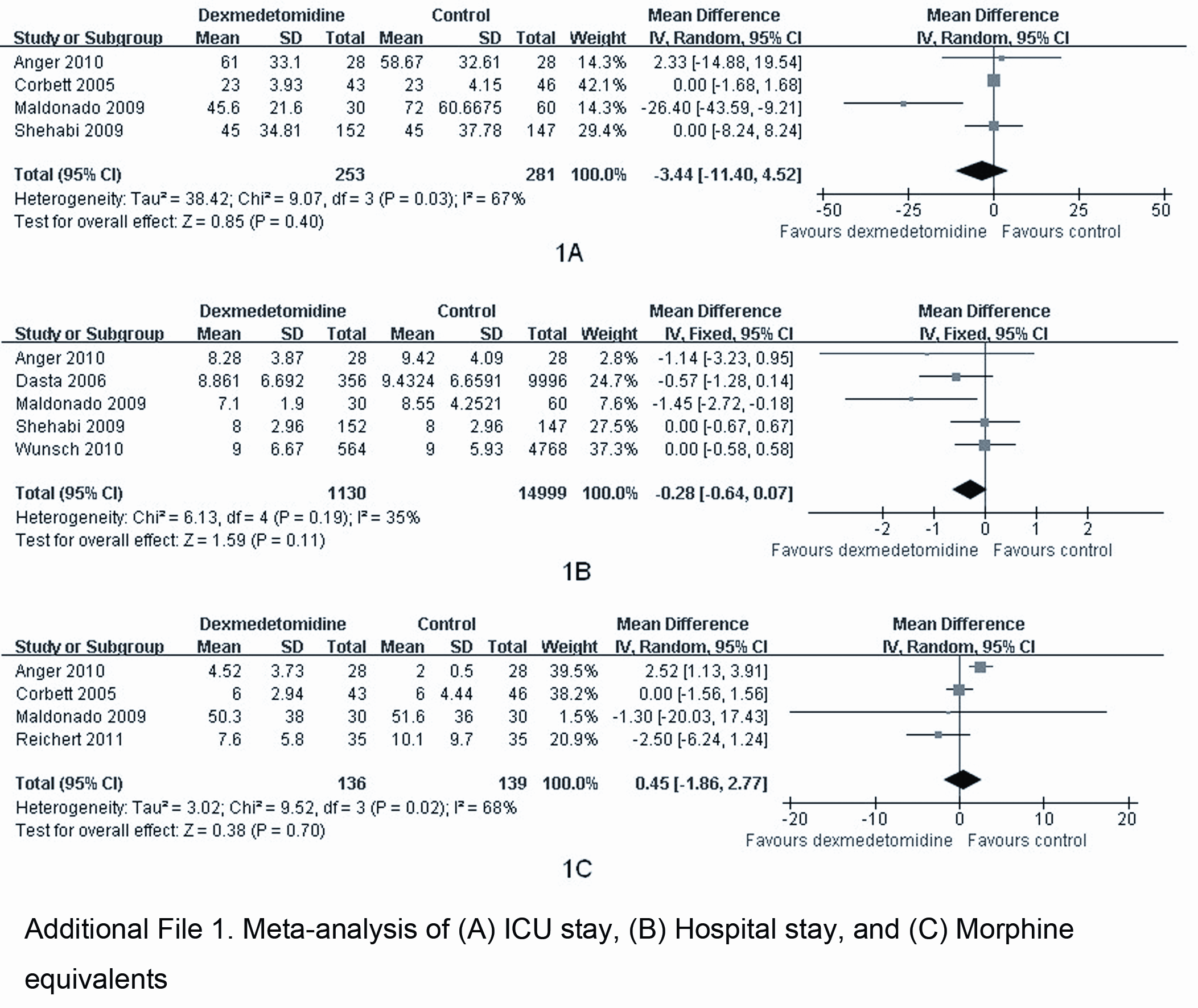

Supplement: Additional File 1 — Figure showing meta-analysis of (A) ICU stay, (B) hospital stay, and (C) morphine equivalents. [file cc11646-S1.PNG]

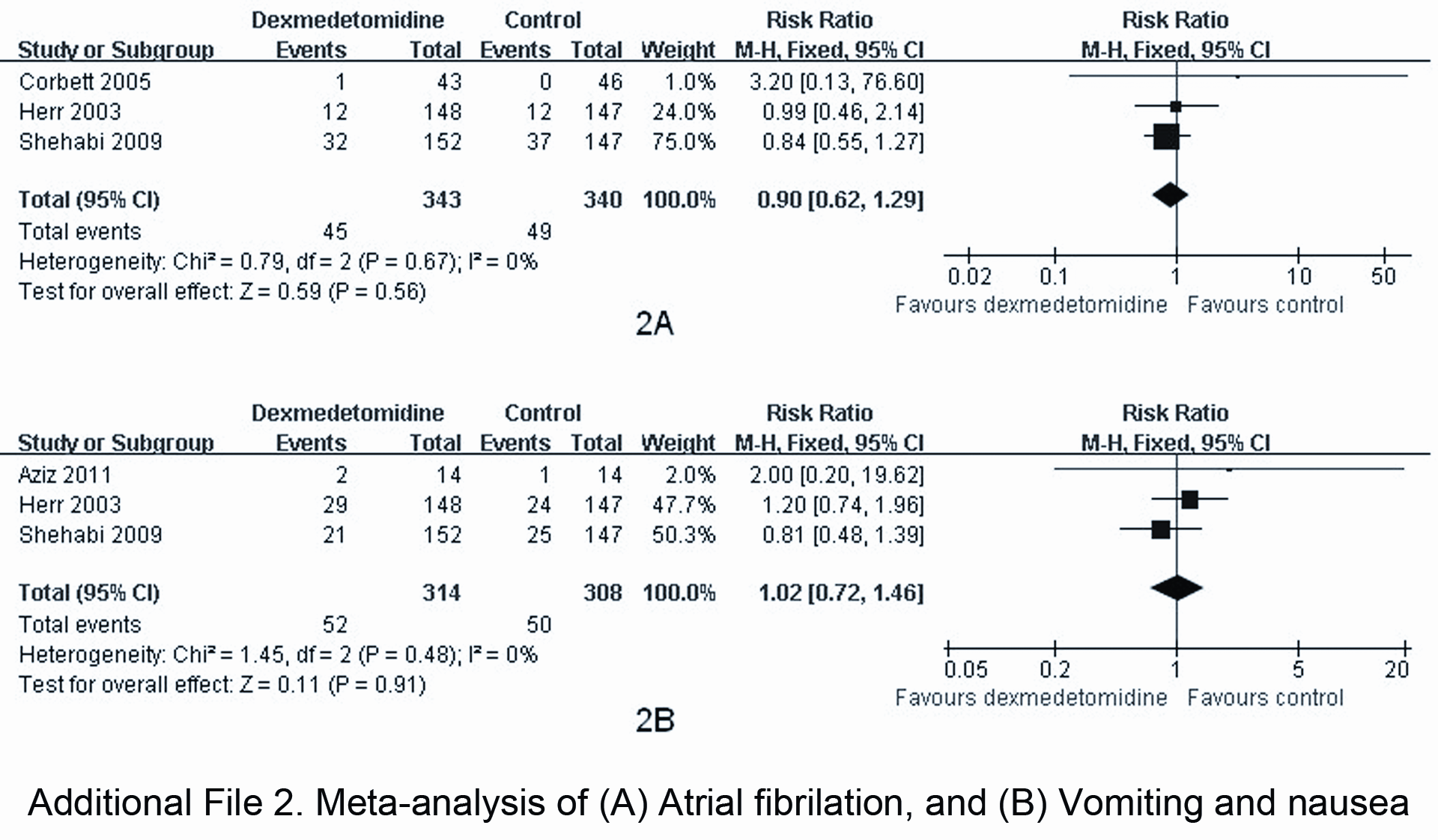

Supplement: Additional File 2 — Figure showing meta-analysis of (A) atrial fibrilation, and (B) vomiting and nausea. [file cc11646-S2.PNG]

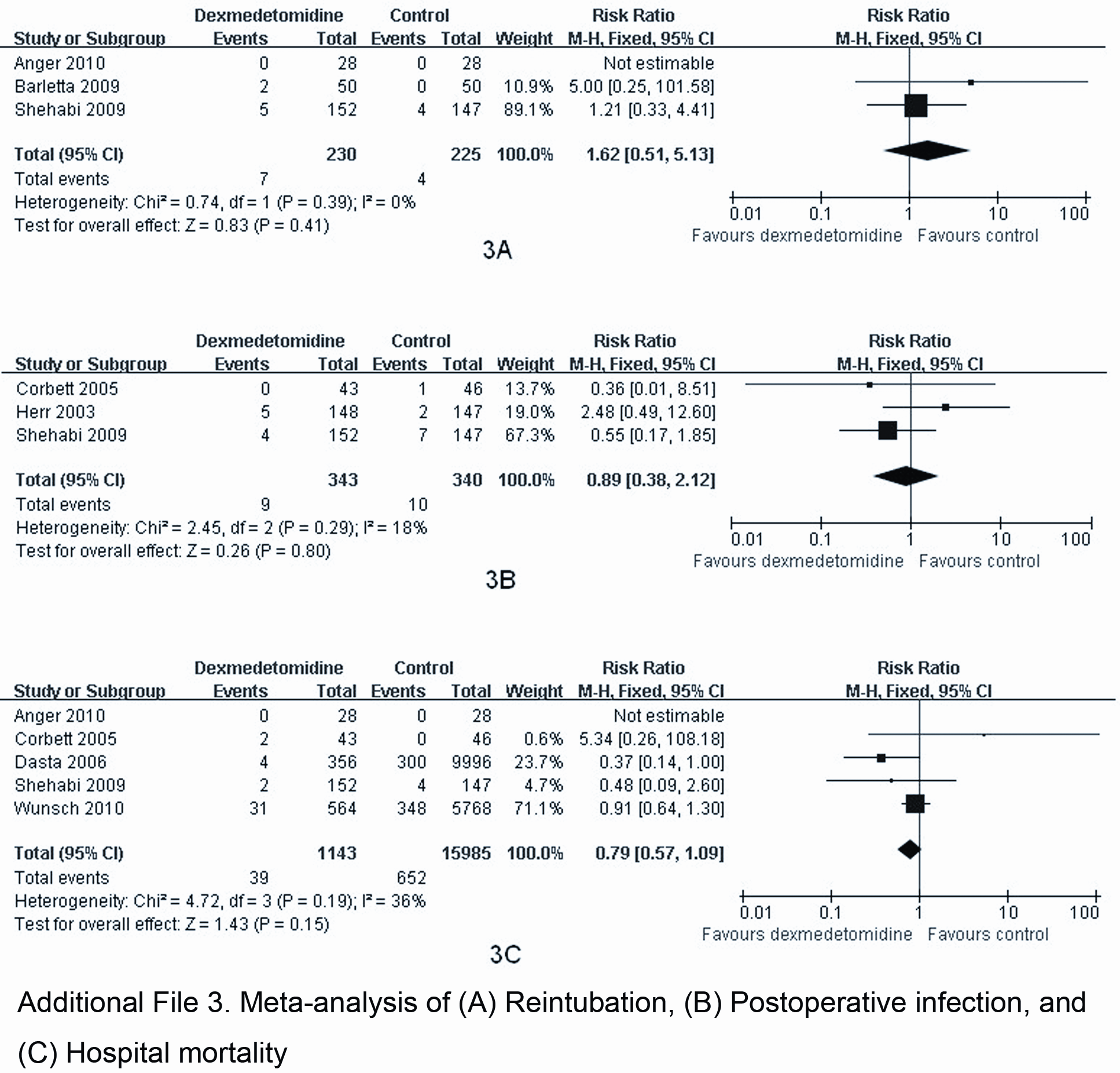

Supplement: Additional File 3 — Figure showing meta-analysis of (A) reintubation, (B) postoperative infection, and (C) hospital mortality. [file cc11646-S3.PNG]
